# Supplementary material for: Molecular epidemiology of Kaposi sarcoma virus in Spain
Source: PLoS One. 2022 Oct 25;17(10):e0274058. doi: 10.1371/journal.pone.0274058 (PMC9595507; doi:10.1371/journal.pone.0274058)

**S2 Fig. 1-11. Alignment of subtype sequences from clinical samples and their corresponding reference sequences.**

**Fig 1**. Alignment of A1 sequences from clinical samples and their reference sequences.


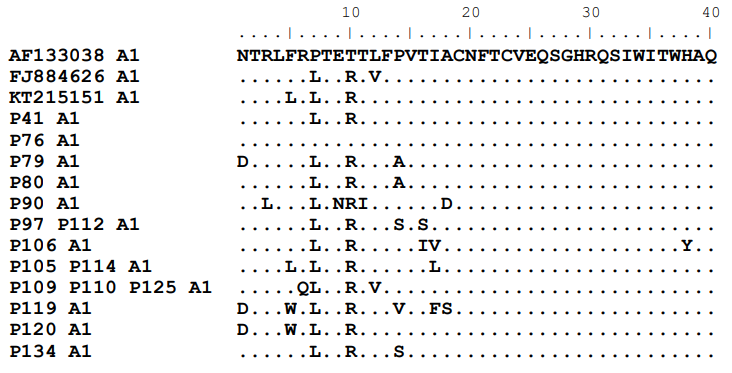


**Fig 2**. Alignment of A2 sequences from clinical samples and their reference sequences.


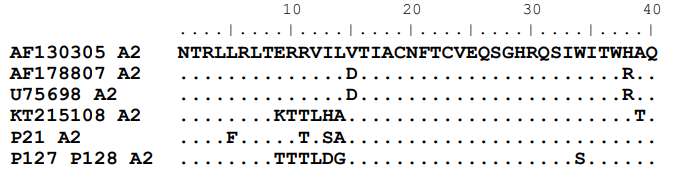


**Fig 3**. Alignment of A3 sequences from clinical samples and their reference sequences.


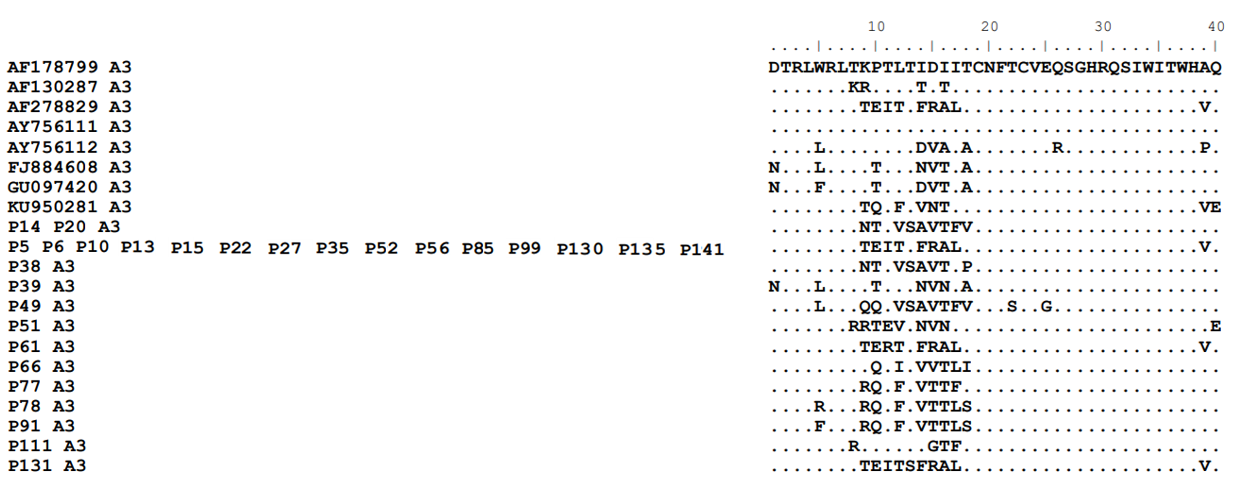


**Fig 4**. Alignment of A4 sequences from clinical samples and their reference sequences.


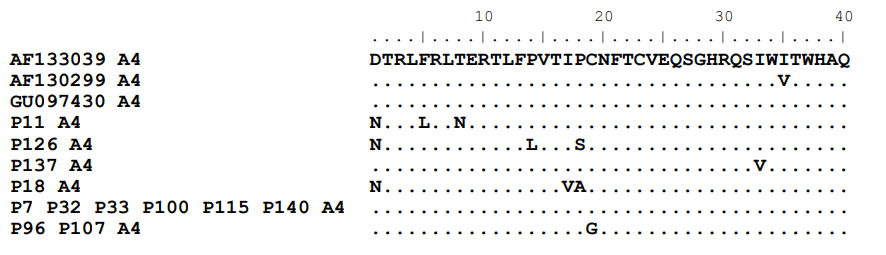


**Fig 5**. Alignment of A5 sequences from clinical samples and their reference sequences.


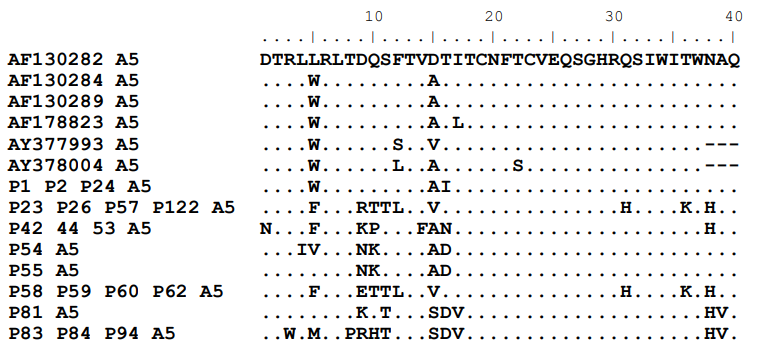


**Fig 6**. Alignment of B1 sequences from clinical samples and their reference sequences.


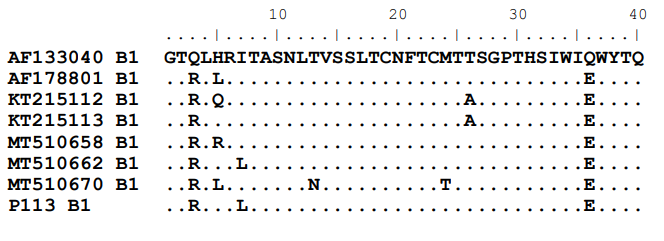


**Fig 7**. Alignment of C1 sequences from clinical samples and their reference sequences.


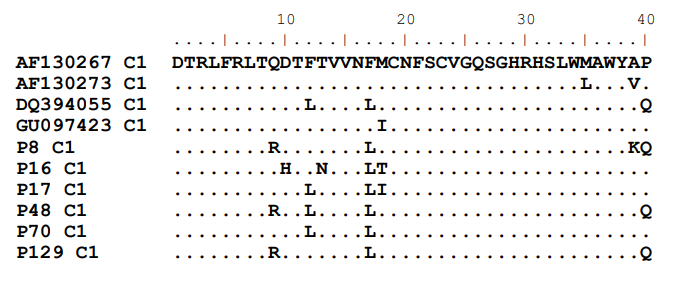


**Fig 8**. Alignment of C2 sequences from clinical samples and their reference sequences.


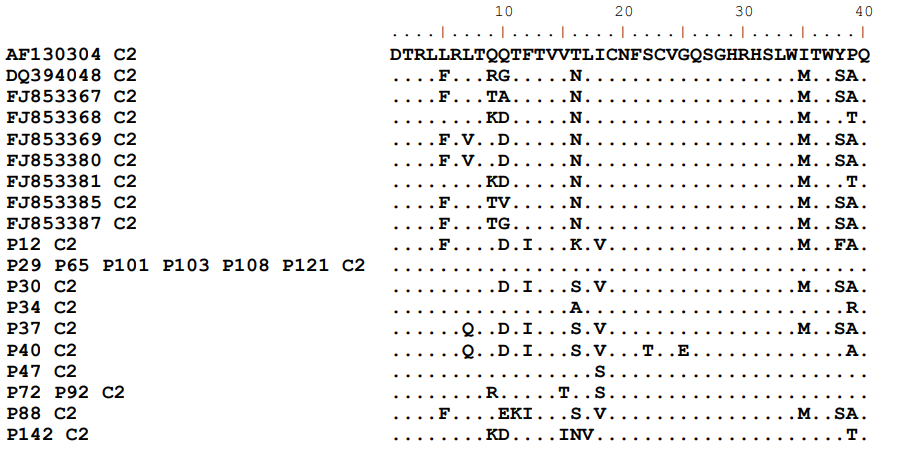


**Fig 9**. Alignment of C3 sequences from clinical samples and their reference sequences.


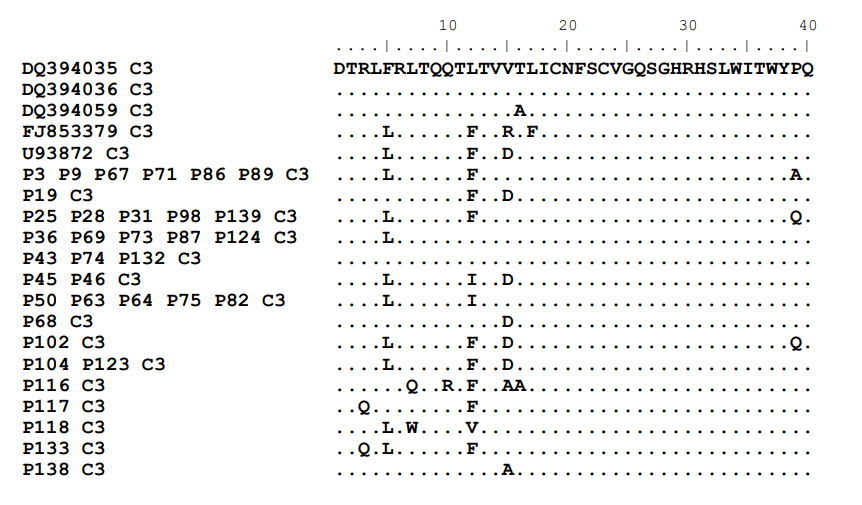


**Fig 10**. Alignment of E1 sequences from clinical samples and their reference sequences.


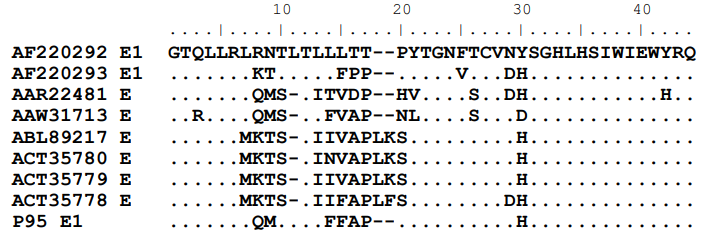


**Fig 11**. Alignment of E2 sequences from clinical samples and their reference sequences.


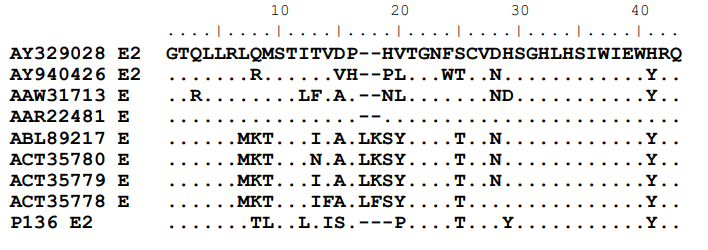

Supplement: S1 File — (DOCX) [file pone.0274058.s001.docx]
