# Supplementary material for: Molecular epidemiology of Kaposi sarcoma virus in Spain
Source: PLoS One. 2022 Oct 25;17(10):e0274058. doi: 10.1371/journal.pone.0274058 (PMC9595507; doi:10.1371/journal.pone.0274058)
Supplement: S1 Table — (DOCX) [file pone.0274058.s002.docx]

**S1 Table**. Detailed information for each patient regarding date of request, age, native country hospital of origin and subtype.

| **ID** | **Year of request** | **Age's patient** | **Country of birth** | **Region** | **Subtype** |
| --- | --- | --- | --- | --- | --- |
| P1 | 2013 | 68 | Spain | Madrid | A5 |
| P2 | 2013 | 38 | Argentina | Madrid | A5 |
| P3 | 2013 | 81 | Spain | Valencia | C3 |
| P4 | 2014 | 61 | Spain | Andalusia | C7 |
| P5 | 2014 | Unknown | Unknown | Navarre | A3 |
| P6 | 2014 | 49 | Spain | Galicia | A3 |
| P7 | 2014 | 49 | Spain | Galicia | A4 |
| P8 | 2014 | 73 | Spain | Galicia | C1 |
| P9 | 2014 | 86 | Spain | Castilla y León | C3 |
| P10 | 2014 | 57 | Unknown | Madrid | A3 |
| P11 | 2014 | 56 | Spain | Andalusia | A4 |
| P12 | 2014 | 71 | Spain | Madrid | C2 |
| P13 | 2014 | 57 | Unknown | Madrid | A3 |
| P14 | 2014 | 72 | Spain | Basque Country | A3 |
| P15 | 2014 | 46 | Unknown | Madrid | A3 |
| P16 | 2014 | 61 | Unknown | Madrid | C1 |
| P17 | 2014 | 89 | Spain | Valencia | C1 |
| P18 | 2017 | 79 | Spain | Castilla y León | A1 |
| P19 | 2014 | 53 | Unknown | Madrid | C3 |
| P20 | 2014 | 28 | Unknown | Madrid | A3 |
| P21 | 2014 | 40 | Spain | Andalusia | A2 |
| P22 | 2014 | 29 | Unknown | Madrid | A3 |
| P23 | 2015 | 33 | Cameroon | Madrid | A5 |
| P24 | 2015 | 44 | Nigeria | Madrid | A5 |
| P25 | 2015 | 89 | Spain | Aragon | C3 |
| P26 | 2015 | 58 | Spain | Madrid | A5 |
| P27 | 2015 | 51 | Spain | Madrid | A3 |
| P28 | 2015 | 79 | Spain | Madrid | C3 |
| P29 | 2015 | 67 | Unknown | Madrid | C2 |
| P30 | 2015 | 60 | Spain | Madrid | C2 |
| P31 | 2015 | 79 | Spain | Madrid | C3 |
| P32 | 2015 | 37 | Spain | Madrid | A4 |
| P33 | 2015 | 36 | Spain | Madrid | A4 |
| P34 | 2015 | 54 | Unknown | Navarre | C2 |
| P35 | 2015 | 86 | Unknown | Madrid | A3 |
| P36 | 2015 | 33 | Unknown | Madrid | C3 |
| P37 | 2015 | 67 | Unknown | Galicia | C2 |
| P38 | 2015 | 46 | Unknown | Madrid | A3 |
| P39 | 2015 | 59 | Spain | Valencia | A3 |
| P40 | 2016 | 67 | Unknown | Galicia | C2 |
| P41 | 2016 | 54 | Spain | Castilla-La Mancha | A1 |
| P42 | 2016 | 48 | Nigeria | Madrid | A5 |
| P43 | 2016 | 22 | Unknown | Catalonia | C3 |
| P44 | 2016 | 28 | South-América | Madrid | A5 |
| P45 | 2016 | 59 | Spain | Cantabria | C3 |
| P46 | 2016 | 59 | Spain | Cantabria | C3 |
| P47 | 2016 | 36 | Spain | Cantabria | C2 |
| P48 | 2016 | 49 | Unknown | Valencia | C1 |
| P49 | 2017 | 5 | Unknown | Madrid | A3 |
| P50 | 2017 | 43 | Spain | Basque Country | C3 |
| P51 | 2017 | 65 | Spain | Madrid | A3 |
| P52 | 2017 | 22 | Unknown | Catalonia | A3 |
| P53 | 2017 | 48 | Nigeria | Madrid | A5 |
| P54 | 2017 | Unknown | Spain | Madrid | A5 |
| P55 | 2017 | 50 | Equatorial Guinea | Madrid | A5 |
| P56 | 2017 | 84 | Spain | Galicia | A3 |
| P57 | 2017 | 46 | Spain | Madrid | A5 |
| P58 | 2017 | 73 | Spain | Aragon | A5 |
| P59 | 2017 | 28 | Colombia | Madrid | A5 |
| P60 | 2017 | 44 | Nigeria | Madrid | A5 |
| P61 | 2017 | 37 | Spain | Madrid | A3 |
| P62 | 2021 | 84 | Spain | Madrid | A5 |
| P63 | 2017 | 43 | Spain | Basque Country | C3 |
| P64 | 2017 | 58 | Unknown | Basque Country | C3 |
| P65 | 2017 | 84 | Spain | Galicia | C2 |
| P66 | 2017 | 47 | Spain | Madrid | A3 |
| P67 | 2017 | 43 | Spain | Basque Country | C3 |
| P68 | 2017 | 46 | Spain | Extremadura | C3 |
| P69 | 2017 | 53 | Spain | Madrid | C3 |
| P70 | 2018 | 95 | Spain | Castilla y León | C1 |
| P71 | 2018 | 91 | Spain | Madrid | C3 |
| P72 | 2018 | 43 | Equatorial Guinea | Madrid | C2 |
| P73 | 2018 | 92 | Unknown | Madrid | C3 |
| P74 | 2018 | 42 | Unknown | Madrid | C3 |
| P75 | 2018 | 43 | Spain | Basque Country | C3 |
| P76 | 2018 | 33 | Unknown | Madrid | A1 |
| P77 | 2018 | 22 | Unknown | Catalonia | A3 |
| P78 | 2018 | 42 | Unknown | Madrid | A3 |
| P79 | 2018 | 87 | Spain | Castilla y León | A1 |
| P80 | 2018 | 53 | Unknown | Madrid | A4 |
| P81 | 2018 | 19 | Morocco | Madrid | A5 |
| P82 | 2018 | 43 | Spain | Basque Country | C3 |
| P83 | 2018 | 3 | Spain | Castilla-La Mancha | A5 |
| P84 | 2018 | 19 | Morocco | Madrid | A5 |
| P85 | 2018 | 88 | Unknown | Madrid | A3 |
| P86 | 2018 | 86 | Spain | Castilla y León | C3 |
| P87 | 2018 | 33 | Spain | Madrid | C3 |
| P88 | 2018 | 83 | Spain | Madrid | C2 |
| P89 | 2019 | 86 | Spain | Castilla y León | C3 |
| P90 | 2019 | 31 | Moldova | Asturias | A1 |
| P91 | 2019 | 59 | Spain | Madrid | A3 |
| P92 | 2019 | 54 | Unknown | Madrid | C2 |
| P93 | 2019 | 36 | Unknown | Andalusia | C7 |
| P94 | 2019 | 43 | Spain | Balearic Islands | A5 |
| P95 | 2019 | 31 | Cuba | Madrid | E1 |
| P96 | 2019 | 44 | Spain | Andalusia | A4 |
| P97 | 2019 | 41 | Unknown | Madrid | A1 |
| P98 | 2019 | 31 | Unknown | Madrid | C3 |
| P99 | 2019 | 44 | Unknown | Madrid | A3 |
| P100 | 2019 | 46 | Spain | Galicia | A4 |
| P101 | 2019 | 59 | Spain | Cantabria | C2 |
| P102 | 2019 | 45 | Unknown | Balearic Islands | C3 |
| P103 | 2019 | 83 | Unknown | Madrid | C2 |
| P104 | 2019 | 59 | Spain | Basque Country | C3 |
| P105 | 2019 | 27 | Unknown | Madrid | A1 |
| P106 | 2019 | 43 | El Salvador | Basque Country | A1 |
| P107 | 2019 | 44 | Spain | Andalusia | A4 |
| P108 | 2019 | 78 | Spain | Andalusia | C2 |
| P109 | 2019 | 27 | Honduras | Madrid | A1 |
| P110 | 2019 | 48 | Spain | Madrid | A1 |
| P111 | 2019 | 56 | Unknown | Madrid | A3 |
| P112 | 2019 | 41 | Unknown | Madrid | A1 |
| P113 | 2020 | 29 | Mexico | Madrid | B1 |
| P114 | 2020 | 27 | Unknown | Madrid | A1 |
| P115 | 2020 | 31 | Spain | Basque Country | A4 |
| P116 | 2020 | 47 | Spain | Canary Islands | C3 |
| P117 | 2020 | 91 | Spain | Madrid | C3 |
| P118 | 2020 | 48 | Unknown | Madrid | C3 |
| P119 | 2020 | 53 | Spain | Madrid | A1 |
| P120 | 2020 | 20 | Unknown | Valencia | A1 |
| P121 | 2020 | 81 | Spain | Castilla-La Mancha | C2 |
| P122 | 2021 | 39 | Spain | Galicia | A5 |
| P123 | 2021 | 85 | Spain | Basque Country | C3 |
| P124 | 2021 | 71 | Spain | Valencia | C3 |
| P125 | 2021 | 33 | Unknown | Madrid | A1 |
| P126 | 2021 | 27 | Honduras | Madrid | A4 |
| P127 | 2021 | 24 | Venezuela | Madrid | A2 |
| P128 | 2021 | 24 | Venezuela | Madrid | A2 |
| P129 | 2021 | 50 | Unknown | Madrid | C1 |
| P130 | 2021 | 74 | Spain | Madrid | A3 |
| P131 | 2021 | 47 | Spain | Basque Country | A3 |
| P132 | 2021 | 42 | Unknown | Madrid | C3 |
| P133 | 2021 | 55 | Spain | Basque Country | C3 |
| P134 | 2021 | 39 | Unknown | Galicia | A1 |
| P135 | 2021 | 61 | Unknown | Castilla y León | A3 |
| P136 | 2021 | 38 | Romania | Madrid | E2 |
| P137 | 2021 | 35 | Spain | Andalusia | A4 |
| P138 | 2021 | 36 | Spain | Andalusia | C3 |
| P139 | 2021 | 54 | Spain | Andalusia | C3 |
| P140 | 2021 | 38 | Unknown | Andalusia | A4 |
| P141 | 2021 | 42 | Spain | Andalusia | A3 |
| P142 | 2021 | 49 | Unknown | Basque Country | C2 |
