# Supplementary material for: Molecular epidemiology of Kaposi sarcoma virus in Spain
Source: PLoS One. 2022 Oct 25;17(10):e0274058. doi: 10.1371/journal.pone.0274058 (PMC9595507; doi:10.1371/journal.pone.0274058)
Supplement: S2 Table — (DOCX) [file pone.0274058.s003.docx]

**S2 Table**. Number and proportion of detected subtypes of HHV-8 in the different regions of Spain during the period 2013-2021.

|  | | | Subtype | | | | | | | | | | | | Total |
| --- | --- | --- | --- | --- | --- | --- | --- | --- | --- | --- | --- | --- | --- | --- | --- |
|  |  |  | A1 | A2 | A3 | A4 | A5 | B1 | C1 | C2 | C3 | C7 | E1 | E2 |  |
| Region | Andalusia | Number | 0 | 1 | 1 | 5 | 0 | 0 | 0 | 1 | 2 | 2 | 0 | 0 | 12 |
|  |  | % within region | 0,0% | 8,3% | 8,3% | 41,7% | 0,0% | 0,0% | 0,0% | 8,3% | 16,7% | 16,7% | 0,0% | 0,0% | 100,0% |
|  | Aragon | Number | 0 | 0 | 0 | 0 | 1 | 0 | 0 | 0 | 1 | 0 | 0 | 0 | 2 |
|  |  | % within region | 0,0% | 0,0% | 0,0% | 0,0% | 50,0% | 0,0% | 0,0% | 0,0% | 50,0% | 0,0% | 0,0% | 0,0% | 100,0% |
|  | Asturias | Number | 1 | 0 | 0 | 0 | 0 | 0 | 0 | 0 | 0 | 0 | 0 | 0 | 1 |
|  |  | % within region | 100,0% | 0,0% | 0,0% | 0,0% | 0,0% | 0,0% | 0,0% | 0,0% | 0,0% | 0,0% | 0,0% | 0,0% | 100,0% |
|  | Canary Islands | Number | 0 | 0 | 0 | 0 | 0 | 0 | 0 | 0 | 1 | 0 | 0 | 0 | 1 |
|  |  | % within region | 0,0% | 0,0% | 0,0% | 0,0% | 0,0% | 0,0% | 0,0% | 0,0% | 100,0% | 0,0% | 0,0% | 0,0% | 100,0% |
|  | Cantabria | Number | 0 | 0 | 0 | 0 | 0 | 0 | 0 | 2 | 2 | 0 | 0 | 0 | 4 |
|  |  | % within region | 0,0% | 0,0% | 0,0% | 0,0% | 0,0% | 0,0% | 0,0% | 50,0% | 50,0% | 0,0% | 0,0% | 0,0% | 100,0% |
|  | Castilla y León | Number | 2 | 0 | 1 | 0 | 0 | 0 | 1 | 0 | 3 | 0 | 0 | 0 | 7 |
|  |  | % within region | 28,6% | 0,0% | 14,3% | 0,0% | 0,0% | 0,0% | 14,3% | 0,0% | 42,9% | 0,0% | 0,0% | 0,0% | 100,0% |
|  | Castilla-La Mancha | Number | 1 | 0 | 0 | 0 | 1 | 0 | 0 | 1 | 0 | 0 | 0 | 0 | 3 |
|  |  | % within region | 33,3% | 0,0% | 0,0% | 0,0% | 33,3% | 0,0% | 0,0% | 33,3% | 0,0% | 0,0% | 0,0% | 0,0% | 100,0% |
|  | Catalonia | Number | 0 | 0 | 2 | 0 | 0 | 0 | 0 | 0 | 1 | 0 | 0 | 0 | 3 |
|  |  | % within region | 0,0% | 0,0% | 66,7% | 0,0% | 0,0% | 0,0% | 0,0% | 0,0% | 33,3% | 0,0% | 0,0% | 0,0% | 100,0% |
|  | Valencia | Number | 1 | 0 | 1 | 0 | 0 | 0 | 2 | 0 | 2 | 0 | 0 | 0 | 6 |
|  |  | % within region | 16,7% | 0,0% | 16,7% | 0,0% | 0,0% | 0,0% | 33,3% | 0,0% | 33,3% | 0,0% | 0,0% | 0,0% | 100,0% |
|  | Extremadura | Number | 0 | 0 | 0 | 0 | 0 | 0 | 0 | 0 | 1 | 0 | 0 | 0 | 1 |
|  |  | % within region | 0,0% | 0,0% | 0,0% | 0,0% | 0,0% | 0,0% | 0,0% | 0,0% | 100,0% | 0,0% | 0,0% | 0,0% | 100,0% |
|  | Galicia | Number | 1 | 0 | 2 | 2 | 1 | 0 | 1 | 3 | 0 | 0 | 0 | 0 | 10 |
|  |  | % within region | 10,0% | 0,0% | 20,0% | 20,0% | 10,0% | 0,0% | 10,0% | 30,0% | 0,0% | 0,0% | 0,0% | 0,0% | 100,0% |
|  | Balearic Islands | Number | 0 | 0 | 0 | 0 | 1 | 0 | 0 | 0 | 1 | 0 | 0 | 0 | 2 |
|  |  | % within region | 0,0% | 0,0% | 0,0% | 0,0% | 50,0% | 0,0% | 0,0% | 0,0% | 50,0% | 0,0% | 0,0% | 0,0% | 100,0% |
|  | Madrid | Number | 9 | 2 | 18 | 4 | 16 | 1 | 2 | 7 | 13 | 0 | 1 | 1 | 74 |
|  |  | % within region | 12,2% | 2,7% | 24,3% | 5,4% | 21,6% | 1,4% | 2,7% | 9,5% | 17,6% | 0,0% | 1,4% | 1,4% | 100,0% |
|  | Navarra | Number | 0 | 0 | 1 | 0 | 0 | 0 | 0 | 1 | 0 | 0 | 0 | 0 | 2 |
|  |  | % within region | 0,0% | 0,0% | 50,0% | 0,0% | 0,0% | 0,0% | 0,0% | 50,0% | 0,0% | 0,0% | 0,0% | 0,0% | 100,0% |
|  | Basque Country | Number | 1 | 0 | 2 | 1 | 0 | 0 | 0 | 1 | 9 | 0 | 0 | 0 | 14 |
|  |  | % within region | 7,1% | 0,0% | 14,3% | 7,1% | 0,0% | 0,0% | 0,0% | 7,1% | 64,3% | 0,0% | 0,0% | 0,0% | 100,0% |
| Total | | Number | 16 | 3 | 28 | 12 | 20 | 1 | 6 | 16 | 36 | 2 | 1 | 1 | 142 |
|  |  | % within region | 11,3% | 2,1% | 19,7% | 8,5% | 14,1% | 0,7% | 4,2% | 11,3% | 25,4% | 1,4% | 0,7% | 0,7% | 100,0% |
