# Supplementary material for: Molecular epidemiology of Kaposi sarcoma virus in Spain
Source: PLoS One. 2022 Oct 25;17(10):e0274058. doi: 10.1371/journal.pone.0274058 (PMC9595507; doi:10.1371/journal.pone.0274058)
Supplement: Attachment — Submitted filename: revisores MJM.docx [file pone.0274058.s004.docx]

PONE-D-22-01291

MOLECULAR EPIDEMIOLOGY OF KAPOSI SARCOMA VIRUS IN SPAIN

PLOS ONE

Dear Dr. Tarragó,

Thank you for submitting your manuscript to PLOS ONE. After careful consideration, we feel that it has merit but does not fully meet PLOS ONE’s publication criteria as it currently stands. Therefore, we invite you to submit a revised version of the manuscript that addresses the points raised during the review process.

Please submit your revised manuscript by Apr 09 2022 11:59PM. If you will need more time than this to complete your revisions, please reply to this message or contact the journal office at [plosone@plos.org](mailto:plosone@plos.org). When you're ready to submit your revision, log on to https://www.editorialmanager.com/pone/ and select the 'Submissions Needing Revision' folder to locate your manuscript file.

Please include the following items when submitting your revised manuscript:

A rebuttal letter that responds to each point raised by the academic editor and reviewer(s). You should upload this letter as a separate file labeled 'Response to Reviewers'.

A marked-up copy of your manuscript that highlights changes made to the original version. You should upload this as a separate file labeled 'Revised Manuscript with Track Changes'.

An unmarked version of your revised paper without tracked changes. You should upload this as a separate file labeled 'Manuscript'.

If you would like to make changes to your financial disclosure, please include your updated statement in your cover letter. Guidelines for resubmitting your figure files are available below the reviewer comments at the end of this letter.

If applicable, we recommend that you deposit your laboratory protocols in protocols.io to enhance the reproducibility of your results. Protocols.io assigns your protocol its own identifier (DOI) so that it can be cited independently in the future. For instructions see: <https://journals.plos.org/plosone/s/submission-guidelines#loc-laboratory-protocols>. Additionally, PLOS ONE offers an option for publishing peer-reviewed Lab Protocol articles, which describe protocols hosted on protocols.io. Read more information on sharing protocols at <https://plos.org/protocols?utm_medium=editorial-email&utm_source=authorletters&utm_campaign=protocols>.

We look forward to receiving your revised manuscript.

Kind regards,

Dirk P. Dittmer, Ph.D.
Academic Editor
PLOS ONE

Journal Requirements:

When submitting your revision, we need you to address these additional requirements.

1. Please ensure that your manuscript meets PLOS ONE's style requirements, including those for file naming. The PLOS ONE style templates can be found at 
https://journals.plos.org/plosone/s/file?id=wjVg/PLOSOne_formatting_sample_main_body.pdf and <https://journals.plos.org/plosone/s/file?id=ba62/PLOSOne_formatting_sample_title_authors_affiliations.pdf>. This requirement has been addressed.

2. Please provide additional details regarding participant consent. In the ethics statement in the Methods and online submission information, please ensure that you have specified what type you obtained (for instance, written or verbal, and if verbal, how it was documented and witnessed). If your study included minors, state whether you obtained consent from parents or guardians. If the need for consent was waived by the ethics committee, please include this information.

Once you have amended this/these statement(s) in the Methods section of the manuscript, please add the same text to the “Ethics Statement” field of the submission form (via “Edit Submission”).

For additional information about PLOS ONE ethical requirements for human subjects research, please refer to <http://journals.plos.org/plosone/s/submission-guidelines#loc-human-subjects-research>. This requirement has been addressed.

3. We note that the grant information you provided in the ‘Funding Information’ and ‘Financial Disclosure’ sections do not match.

When you resubmit, please ensure that you provide the correct grant numbers for the awards you received for your study in the ‘Funding Information’ section.

This mistake has been addressed.

4. Thank you for stating the following in the Funding Section of your manuscript: 
"This work was supported by funds and a grant from Instituto de Salud Carlos III. Project code MPY1372/12 and AESI2021 PI21CIII/00011 .The funder had no role in study design, data collection and analysis, decision to publish, or preparation of the manuscript."

We note that you have provided funding information. However, funding information should not appear in the Funding section or other areas of your manuscript. We will only publish funding information present in the Funding Statement section of the online submission form. 
Please remove any funding-related text from the manuscript and let us know how you would like to update your Funding Statement. Currently, your Funding Statement reads as follows: 
"DT. This work was supported by funds and a grant from Instituto de Salud Carlos III. Project code MPY1372/12 and AESI2021 PI21CIII/00011. The funders had no role in study design, data collection and analysis, decision to publish, or preparation of the manuscript."

Please include your amended statements within your cover letter; we will change the online submission form on your behalf.

5. We note that you have stated that you will provide repository information for your data at acceptance. Should your manuscript be accepted for publication, we will hold it until you provide the relevant accession numbers or DOIs necessary to access your data. If you wish to make changes to your Data Availability statement, please describe these changes in your cover letter and we will update your Data Availability statement to reflect the information you provide.

6. Your ethics statement should only appear in the Methods section of your manuscript. If your ethics statement is written in any section besides the Methods, please move it to the Methods section and delete it from any other section. Please ensure that your ethics statement is included in your manuscript, as the ethics statement entered into the online submission form will not be published alongside your manuscript.

This requirement has been addressed

7. We note that Figure 3 in your submission contain map/satellite images which may be copyrighted. All PLOS content is published under the Creative Commons Attribution License (CC BY 4.0), which means that the manuscript, images, and Supporting Information files will be freely available online, and any third party is permitted to access, download, copy, distribute, and use these materials in any way, even commercially, with proper attribution. For these reasons, we cannot publish previously copyrighted maps or satellite images created using proprietary data, such as Google software (Google Maps, Street View, and Earth). For more information, see our copyright guidelines: http://journals.plos.org/plosone/s/licenses-and-copyright.
 
We require you to either (1) present written permission from the copyright holder to publish these figures specifically under the CC BY 4.0 license, or (2) remove the figures from your submission:
 
a. You may seek permission from the original copyright holder of Figure 3 to publish the content specifically under the CC BY 4.0 license.  
 
We recommend that you contact the original copyright holder with the Content Permission Form (http://journals.plos.org/plosone/s/file?id=7c09/content-permission-form.pdf) and the following text:
“I request permission for the open-access journal PLOS ONE to publish XXX under the Creative Commons Attribution License (CCAL) CC BY 4.0 (http://creativecommons.org/licenses/by/4.0/). Please be aware that this license allows unrestricted use and distribution, even commercially, by third parties. Please reply and provide explicit written permission to publish XXX under a CC BY license and complete the attached form.”
 
Please upload the completed Content Permission Form or other proof of granted permissions as an ""Other"" file with your submission.
 
In the figure caption of the copyrighted figure, please include the following text: “Reprinted from [ref] under a CC BY license, with permission from [name of publisher], original copyright [original copyright year].”
 
b. If you are unable to obtain permission from the original copyright holder to publish these figures under the CC BY 4.0 license or if the copyright holder’s requirements are incompatible with the CC BY 4.0 license, please either i) remove the figure or ii) supply a replacement figure that complies with the CC BY 4.0 license. Please check copyright information on all replacement figures and update the figure caption with source information. If applicable, please specify in the figure caption text when a figure is similar but not identical to the original image and is therefore for illustrative purposes only.
The following resources for replacing copyrighted map figures may be helpful:
 
USGS National Map Viewer (public domain): http://viewer.nationalmap.gov/viewer/
The Gateway to Astronaut Photography of Earth (public domain): http://eol.jsc.nasa.gov/sseop/clickmap/
Maps at the CIA (public domain): https://www.cia.gov/library/publications/the-world-factbook/index.html and https://www.cia.gov/library/publications/cia-maps-publications/index.html
NASA Earth Observatory (public domain): http://earthobservatory.nasa.gov/
Landsat: http://landsat.visibleearth.nasa.gov/
USGS EROS (Earth Resources Observatory and Science (EROS) Center) (public domain): http://eros.usgs.gov/#
Natural Earth (public domain): <http://www.naturalearthdata.com/>

This requirement has been addressed.

8. Please include captions for your Supporting Information files at the end of your manuscript, and update any in-text citations to match accordingly. Please see our Supporting Information guidelines for more information: http://journals.plos.org/plosone/s/supporting-information.

[Note: HTML markup is below. Please do not edit.]
This requirement has been addressed.
Reviewers' comments:

Reviewer's Responses to Questions

Comments to the Author

1. Is the manuscript technically sound, and do the data support the conclusions?

The manuscript must describe a technically sound piece of scientific research with data that supports the conclusions. Experiments must have been conducted rigorously, with appropriate controls, replication, and sample sizes. The conclusions must be drawn appropriately based on the data presented.

Reviewer #1: Partly

The manuscript has been rewritten acordingly with the new data obtained from phylogenetic analysis

2. Has the statistical analysis been performed appropriately and rigorously?

Reviewer #1: No

Statistical analysis has been performed taking into consideration a previous study by Oliveira et al which analysed variables such as viral load and other variables presented in the currrent study. A. de Oliveira Lopes, N. Spitz, C.R. de S. Reis, V.S. de Paula, Update of the global distribution of human gammaherpesvirus 8 genotypes, Sci. Reports 2021 111. 11 (2021) 1-9. <https://doi.org/10.1038/s41598-021-87038-9>

3. Have the authors made all data underlying the findings in their manuscript fully available?

The [PLOS Data policy](http://www.plosone.org/static/policies.action#sharing) requires authors to make all data underlying the findings described in their manuscript fully available without restriction, with rare exception (please refer to the Data Availability Statement in the manuscript PDF file). The data should be provided as part of the manuscript or its supporting information, or deposited to a public repository. For example, in addition to summary statistics, the data points behind means, medians and variance measures should be available. If there are restrictions on publicly sharing data—e.g. participant privacy or use of data from a third party—those must be specified.

Reviewer #1: No

All sequence data have been uploaded to GenBank. Therefore we obtained 142 accesion numbers corresponding to 142 sequences yielded in the current study.

4. Is the manuscript presented in an intelligible fashion and written in standard English?

PLOS ONE does not copyedit accepted manuscripts, so the language in submitted articles must be clear, correct, and unambiguous. Any typographical or grammatical errors should be corrected at revision, so please note any specific errors here.

Reviewer #1: No

The manuscript has been corrected by an American scientis working in the microbiology field.

5. Review Comments to the Author

Please use the space provided to explain your answers to the questions above. You may also include additional comments for the author, including concerns about dual publication, research ethics, or publication ethics. (Please upload your review as an attachment if it exceeds 20,000 characters)

Reviewer #1: Review of manuscript “Molecular Epidemiology of Kaposi sarcoma virus in Spain” by Gómez I. et al.

Summary
The study describes the analysis of 142 KSHV K1 subtypes, circulating in Spain between 2013-2021. As pointed out by the authors, not many KSHV genotyping studies have been published from Spain, so this is a welcome report for KSHV researchers. The majority of KSHV K1 subtypes identified were A and C as would be expected in Europe, however, rare subtypes E and F were also found. In particular, the E1 subtype discovery is interesting as this KSHV K1 subtype has not been reported in Europe previously to my knowledge.

The authors also report a variant of the A3 subtype that has a unique series of eight polymorphisms. In addition, variation geographically in K1 subtype distribution was not noted in Spain as has been reported for other European countries like Italy.

Major Concerns

The primary major concern is the lack of phylogenetic analysis and a phylogenetic tree. An evolutionary analysis of the study sequences visualized as a phylogenetic tree, for instance using IQTree or BEAST software, would be a necessary addition to the paper. While the amino acid alignments are informative, the evolutionary relationships between the individual subtypes are lost. As mentioned, to date the E1 subtype has only been found in indigenous peoples from South America. More recently, studies from Cuba have identified an E2 subtype that is similar to but distinct from the original E. A phylogenetic tree with both E1 and E2 references would help inform readers as to the evolutionary relationship of the E subtype from the current study, particularly as ethnicity information is not known for the individual. A similar argument could be made for the three F subtypes. It would be interesting to understand the relationship of the current study F subtypes with the F2 reported from French studies. Lastly, the A3 polymorphisms described in 16 of 19 sequences would be best visualized in a phylogenetic tree analysis to better understand the evolutionary distances within the A3 branch as well as the contribution the newly described sequences provide to the KSHV A genotype overall.

In results section a new subheading has been added which includes phylogenetic trees with all our K1 sequences (142) and all mentioned above by the reviewer (101 sequences). Moreover, all this suggestions have been added to discussion
The authors should highlight further in the discussion that they are reporting the first subtype E KSHV in Europe and the additional subtype F in relation to the recent French studies.

This point has been addressed.
In general many of the references are older and more recent highly relevant references such as the JAry el paper describing sub=type F in France should be added.

New references have been added including the reference cited by the reviewer and others concerning E subtypes found in Cuba.
A major concern for the paper is that there are frequent misspellings that detracts from the message. These are easily addressable but would need to be corrected prior to publication.

The manuscript has been revised by a native English speaker working in the microbiology field.

Minor Concerns
It wasn’t clear what is meant by the statement “Only subgenotype C2 showed significant higher viral loads than subgenotype C3”. Does this mean that C3 and C2 subtype samples were observed to have higher viral loads than the other KSHV K1 subtypes found in the study, for instance A3 and A5, but that C2 displayed the highest VL overall? This is later clarified in the results section, lines 241-245, but as a summary point in the abstract needs clarification.
Abstract has a limited number of words and we decided to state only when differences were found. However, this point has been rewritten to clarify it.
KSHV K1 A5, B1, C3 etc. are more commonly referred to as “subtypes” in the literature. Don’t necessarily need to replace “sub-genotype” description with “subtype”, just an observation.
Subgenotype has been replaced by subtype
Jary et al. recently defined the F2 subtype as distinct from F subtypes commonly found in Africa, now referred to as F1. This can be added to the introduction in line 87.
reference 17 has been added
In line 147, it was noted that only three A3 subtypes were used for comparison in the sequence alignments. Did BLAST searches of the newly described A3 indicate homology to any other published KSHV K1 subtype references?

Eight A3 subtype sequences have nbeen added to the study, in total 101 references sequences available at Genbank have been studied
In lines 164-166, it was noted that only one reference was used for comparison for the rare KSHV K1 subtypes. For some, like C6, there are few available, but the sequence comparisons would be strengthened with the addition of more references. The E2 reference sited from the Biggar et al paper would be considered an E1 due to the publication of E subtypes from Cuba (Kouri,V et al AIDS 2007 and Virology 2012). It would be of interest to compare the current study sequence to the Cuban sequences, as previously mentioned, in order to confirm or clarify the E subtype found in Spain. In addition, F2 subtype references should be added from Lacoste,V. Virology 2000 or Jary et al. J. Inf. Diseases 2020 publications.

All these referred sequences have been added to the current study
In lines 225-227, there is some confusion about why the author’s feel that subtype C3 is the most prevalent. The sentence “This could be explained by the fact that the number of subgenotypes obtained for genotype A (A1-A5) was higher than in genotype C (C1, C2, C3 and C5)” doesn’t seem to support why C3 is found more frequently.

This point has been addressed
In section 3.3, could the author’s comment if they think that the availability of samples collected year to year could affect the observations made concerning frequency of subtypes observed?
In sections 3.4 and 3.5, some of the observations in certain categories are based upon small sample sizes which should be discussed as a limitation.

A limitation of study concerning small sampling in certain years has been added to the corresponding section
In the discussion, it was noted that the frequency of KSHV detection did not vary significantly north to south in Spain as has been reported in Italy. Studies in Italy, as well as Uganda, have found associations between malaria transmission and KSHV prevalence. While the authors admit that the study wasn’t designed to address this question, perhaps they could comment on malaria incidence as a risk factor for KSHV in Spain as well as list the social factors referred to in lines 343-345.

This point is discussed in discussion section
In the supplemental alignment tables, not sure what is meant by alignment of “problem” samples? The reference used for alignment is a KSHV A2 subtype. Alignment of sequences that are not A2 would have polymorphisms. Suggest “problem” be removed from the titles or discussed within the manuscript for clarification.

This point has been addressed.
No mention of data availability was found in the manuscript. Will the sequences be submitted to GenBank or some other publicly assessable resource?

All sequence data have been uploaded to GenBank. Therefore we obtained 142 accesion numbers corresponding to 142 sequences yielded in the current study.

Concluding comment
Publications of KSHV sequences from an understudied area would be of interest to the KSHV community. As KSHV K1 subtypes reflect ethnic and geographical differences, the diverse distribution of subtypes noted in the current study highlight that urban centers may have several circulating strains which can vary over time as communities change.

6. PLOS authors have the option to publish the peer review history of their article ([what does this mean?](https://journals.plos.org/plosone/s/editorial-and-peer-review-process#loc-peer-review-history)). If published, this will include your full peer review and any attached files.


If you choose “no”, your identity will remain anonymous but your review may still be made public.


Do you want your identity to be public for this peer review? For information about this choice, including consent withdrawal, please see our [Privacy Policy](https://www.plos.org/privacy-policy).

Reviewer #1: No

[NOTE: If reviewer comments were submitted as an attachment file, they will be attached to this email and accessible via the submission site. Please log into your account, locate the manuscript record, and check for the action link "View Attachments". If this link does not appear, there are no attachment files.]

While revising your submission, please upload your figure files to the Preflight Analysis and Conversion Engine (PACE) digital diagnostic tool, <https://pacev2.apexcovantage.com/>. PACE helps ensure that figures meet PLOS requirements. To use PACE, you must first register as a user. Registration is free. Then, login and navigate to the UPLOAD tab, where you will find detailed instructions on how to use the tool. If you encounter any issues or have any questions when using PACE, please email PLOS at [figures@plos.org](mailto:figures@plos.org). Please note that Supporting Information files do not need this step.

In compliance with data protection regulations, you may request that we remove your personal registration details at any time. [(Remove my information/details)](https://www.editorialmanager.com/pone/login.asp?a=r). Please contact the publication office if you have any questions.
